# The Genome-Wide Identification, Characterization, and Expression Analysis of the Strictosidine Synthase-like Family in Maize (*Zea mays* L.)

**DOI:** 10.3390/ijms241914733

**Published:** 2023-09-29

**Authors:** Lei Gu, Yongyan Cao, Xuanxuan Chen, Hongcheng Wang, Bin Zhu, Xuye Du, Yiyue Sun

**Affiliations:** School of Life Sciences, Guizhou Normal University, Guiyang 550025, China; 201808009@gznu.edu.cn (L.G.); 232100100386@gznu.edu.cn (Y.C.); c18089643218@163.com (X.C.); wanghc@gznu.edu.cn (H.W.); 201703008@gznu.edu.cn (B.Z.); duxuye@gznu.edu.cn (X.D.)

**Keywords:** maize, strictosidine synthase, abiotic/biotic stress, genome-wide analysis

## Abstract

Maize is often subjected to various environmental stresses. The strictosidine synthase-like (SSL) family is thought to catalyze the key step in the monoterpene alkaloids synthesis pathway in response to environmental stresses. However, the role of *ZmSSL* genes in maize growth and development and its response to stresses is unknown. Herein, we undertook the systematic identification and analysis of maize *SSL* genes. Twenty *SSL* genes were identified in the maize genome. Except for chromosomes 3, 5, 6, and 10, they were unevenly distributed on the remaining 6 chromosomes. A total of 105 *SSL* genes from maize, sorghum, rice, *Aegilops tauschii*, and Arabidopsis were divided into five evolutionary groups, and *ZmSSL* gene structures and conserved protein motifs in the same group were similar. A collinearity analysis showed that tandem duplication plays an important role in the evolution of the SSL family in maize, and *ZmSSL* genes share more collinear genes in crops (maize, sorghum, rice, and *Ae. tauschii*) than in Arabidopsis. *Cis*-element analysis in the *ZmSSL* gene promoter region revealed that most genes contained many development and stress response elements. We evaluated the expression levels of *ZmSSL* genes under normal conditions and stress treatments. *ZmSSL4–9* were widely expressed in different tissues and were positively or negatively regulated by heat, cold, and infection stress from *Colletotrichum graminicola* and *Cercospora zeina*. Moreover, ZmSSL4 and ZmSSL5 were localized in the chloroplast. Taken together, we provide insight into the evolutionary relationships of the *ZmSSL* genes, which would be useful to further identify the potential functions of *ZmSSLs* in maize.

## 1. Introduction

Maize is one of the most widely distributed food crops in the world, with the total area under cultivation only less than wheat and rice. In addition to being eaten, it can also be used to produce feed and ethanol. Crops (as for example maize) are often subjected to various natural disasters (abiotic and biotic stress) during their growth and development stages, which have a great impact on their quality and yield [1]. Because plants lack motility, they have evolved complex stress response systems, and a critical step in these responses is signal transduction [1,2]. Many membrane-localized receptors sense stress signals or plant hormones (abscisic acid, ABA; ethylene; salicylic acid, SA; and methyl jasmonate, JA) produced by stress treatments and transmit stress signals into the cells through the influx of Ca^2+^ and activate downstream genes of protein kinases and transcription factors (TFs). This, in turn, activates the expression of relevant defense genes to enhance plant stress tolerance [2,3,4,5,6]. Alkaloids are an important secondary metabolite of plants; their synthesis is regulated by TFs, and they play a significant role in plant resistance to biotic and abiotic stress [7,8]. Strictosidine (STR) is a well-known precursor substance that works on the de novo biosynthesis of many plant alkaloids, and strictosidine synthase (SSL, E.C.4.3.3.2.) catalyzes the synthesis of strictosamide (STR) from geraniol diphosphate and tryptamine in cytoplasm [8,9]. Previous research has shown that chitooligosaccharides, the only positively charged basic amino oligosaccharides in nature, can induce the expression of *STR* and other genes, thereby improving the drought tolerance and yield of plants [10,11,12].

The protein sequences of the plant *SSL* gene family have two conservative motifs: strictosidine synthase (SS) and the SS-like N-terminal domain [8]. The SS motif is responsible for the synthesis of STR, and the fold conformation of the SS-like N-terminal domain is similar to the SS motif [8]. The sequence of the SSL extracellular motif (containing 400 amino acids) also shares a high similarity to the 100 kDa *Drosophila* immune-related protein (i.e., hemomucin, an important receptor) [8,9].

Until now, the *SSL* gene family has been systematically characterized only in *Populus trichocarpa* (13 members) [8], *Arabidopsis thaliana* (15 members) [13], and *Salix purpurea* (22 members) [8]. All Arabidopsis *SSL* members are biotic/abiotic-stress-responsive [9,13]. Further functional analysis revealed that *AtSSL4–7* displays the enzyme activity necessary to catalyze the synthesis of STR, and the AtSSL4–7 gene is upregulated by treatments of SA, JA, and ethylene [13]. The expression level of the *PtrSSL2, 6*, *8*, *10*, and *12* genes is elevated by drought and high salt levels, and the *PtrSSL2*, *7*, *8*, and *10* genes respond to leaf blight (*Alternaria alternata*) stress [8]. In addition to responding to adversity, five or seven *PtrSSL* family members were highly expressed in stems or leaves, respectively [8]. These results imply that *SSL* genes play an important role in normal growth and response to biotic/abiotic stresses in Poplar trees. There are a few reports about the numbers and function of *SSL* genes in other species. In *Catharanthus roseus*, the *SSL* gene is highly induced by drought stress, thus conferring plant drought tolerance [10]. Tomato miR1916 pairs with *STR* gene mRNA and negatively regulates its accumulation; the over-expression of *miR1916* in tomato plants significantly decreased the drought tolerance of transgenic lines [14]. Conversely, the silencing of *miR1916* in *Solanum lycopersicum* increased the expression level of *STR-2*, some immune-response-related proteins, and MYB transcription factor genes, which, in turn, significantly elevated the tolerance of tomato plant leaves to two common fungi (*Phytophthora infestans* and *Botrytis cinerea*) [15]. Overall, although several reports indicate that the *SSL* gene family is related to plant stress responses, the role of *ZmSSL* genes in maize growth and development or response to biotic/abiotic stresses is far from known.

In the present study, we systematically identified and analyzed the maize *SSL* gene family members. With the help of homology comparisons using conserved protein motifs, 20 *ZmSSL* genes were identified; chromosomal localization was also undertaken along with the clarification of phylogenetics and gene structure. *Cis*-elements located in promoter analysis were also carried out. Moreover, we analyzed the expression level of *ZmSSL* genes under normal growth and various biotic/abiotic conditions using published transcriptome data and the RT-qPCR method. This work provides new information about the evolutionary relationships of the *ZmSSL* gene family, which provides a foundation for further detecting their possible biological functions.

## 2. Results

### 2.1. Identification of the Maize SSL Gene Family

To systematically identify the *SSL* gene family members in maize (*Zea mays* L.), sorghum (*Sorghum bicolor* L.), rice (*Oryza sativa* L.), and one of the parents of common wheat, *Aegilops tauschii* (*Aegilops tauschii* Coss.), a homology analysis was conducted in a recent version of the genomic database of each species using AtSSLs protein sequences [8] and two conserved motifs (PF03088 and PF20067) based on the hidden Markov model (HMM) [8]. After taking the intersection of comparison results and removing duplicates, 20, 21, 16, and 33 *SSL* genes were evaluated in maize, sorghum, rice, and *Ae. tauschii*, respectively. All of these identified genes were named in the order of the position in their chromosomes (Supplementary Table S1). The maize *SSL* gene family is unevenly distributed on 6 of the 10 chromosomes with the highest number (10 members) on chromosome 8 and the lowest on chromosomes 4 and 9 (Supplementary Table S1). Like the maize, the other three *SSL* family members were also unevenly distributed (Supplementary Table S1). The sequence characteristics and predicted subcellular localization of the SSL genes in these four species are shown in Table 1 and Supplementary Table S2. The *ZmSSL* genes encode proteins of 197–412 amino acids (aa) in length (average: 340 aa), with isoelectric points (PI) ranging from 5.81 to 8.92 (average: 6.57), and protein molecular weights (Mw) of 20.81–46.54 kDa (average: 36.52). Among the 21 *SbSSL* proteins, the average aa, PI, and Mw were 353, 7.23, and 38.23, respectively. *Ae. tauschii* contains 33 SSL members; the average aa, PI, and Mw were 337, 7.61, and 36.63, respectively. The number of *OsSSLs* was almost identical to Arabidopsis; the average aa, PI, and Mw were 384, 8.00, and 41.66, respectively. Based on the predicted subcellular localization, *ZmSSL* genes were located in the chloroplast/thylakoid (14 members), extracellular/cell wall (4 members), plasma membrane (1 member), and endoplasmic reticulum (1 member), respectively (Table 1). The members of the other four species were also distributed in other places, such as the mitochondrion, vascular membrane, and cytosol (Table 1 and Supplementary Table S2).

### 2.2. Phylogenetic Analysis of the ZmSSL Genes’ Protein

**Figure 1:**
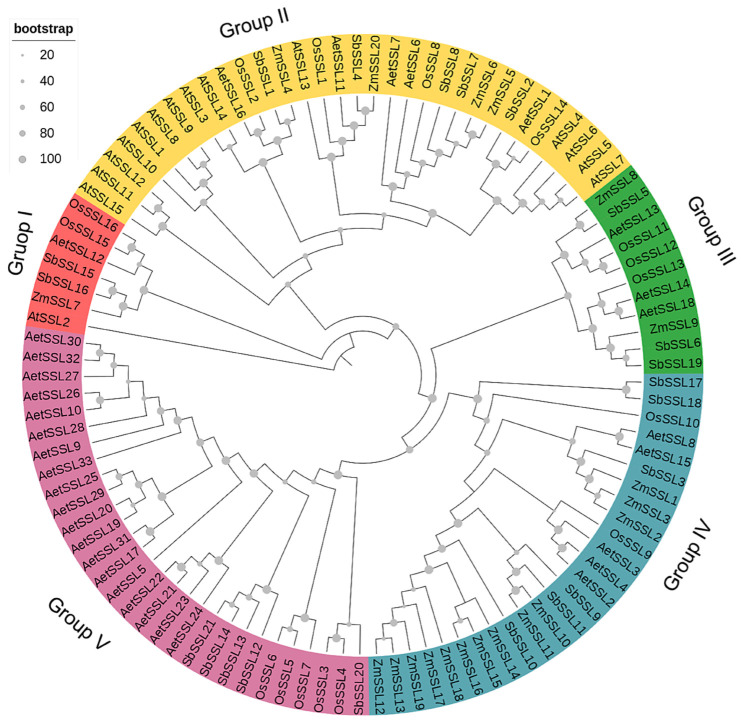
Phylogenetic analysis of SSL proteins in *Zea mays* (Zm), *Sorghum bicolor* (Sb), *Oryza sativa* (Os), *Aegilops tauschii* (Aet), and *Arabidopsis thaliana* (At). The unrooted maximum likelihood (10,000 bootstrap replicates) phylogenetic tree was divided into five groups; different colors are used to distinguish each group.

To examine the evolutionary relationships of *SSL* genes in maize, sorghum, rice, *Ae. tauschii*, and Arabidopsis (model species), a maximum likelihood phylogenetic tree was constructed using the protein sequences of the *SSL* genes listed in Table 1 and Supplementary Table S2. As shown in Figure 1, the 105 *SSL* members from five species could be divided into five groups (I–V), each group including 32, 11, 26, 29, and 7 *SSL* genes, respectively. SSL genes were present in Group I and Group II from all five plants, implying that members in these two groups may undergo a similar evolutionary pattern (Figure 1). Meanwhile, there is also an unequal distribution in the evolutionary tree; all *AtSSLs* were only represented in Groups I and II (14 members in I and 1 member in V) (Figure 1); the *SSL* genes of four crops were mostly represented in Groups III–V (from the same ancestor) (Figure 1), which might be due to the relatively distant evolutionary relationship between Arabidopsis and the crops. Maize *SSL* genes were predominantly in Group IV (13 members), and all branches of *ZmSSL* genes were more adjacent to *SbSSL* gene branches than to the other two crops (Figure 1). This indicates that maize and sorghum are evolutionary more closely related.

### 2.3. Structural Analysis of ZmSSL Genes

**Figure 2:**
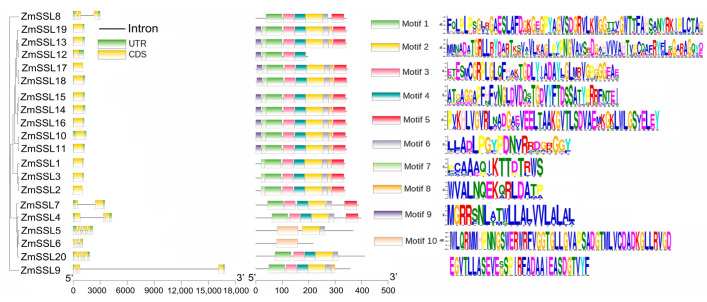
Gene exon–intron structure and conserved protein motifs of *ZmSSL* genes. UTR and CDS are shown as colorful boxes and introns as lines. Ten conserved motifs with common sequences are also shown in differently colored boxes. The clustering of *ZmSSL* genes was based on the phylogenetic tree shown in Figure 1.

To further explore the *ZmSSL* family members, we evaluated the gene structure and conserve motifs of *ZmSSL* genes. The gene exon–intron structure/distribution analysis indicated that, except for *ZmSSL4/5/6/20* (Group II), where the number of exons and introns varies from each other, the same group displays structural similarities; *ZmSSL7* (Group I) contained 2 exons and 1 intron; four Group II members contained 3, 4, or 6 exons and 2, 3, or 5 introns, respectively; two Group III members (*ZmSSL8* and *ZmSSL9*) included 3 exons and 2 introns; and all of Group IV (13 members) contained only 1 exon and no introns (Figure 2). Ten conserve protein motifs (amino acid numbers ranging from 15 to 80) were identified in *ZmSSL* genes using MEME (Figure 2). Further analysis revealed that there were similar sequences and numbers of motifs in the same subgroups (indicating close evolutionary branches), while there were some differences between the different subgroups (Figure 1 and Figure 2). For example, ZmSSL1–3 contained motifs 1–8 (Figure 2). The most conserved motif, motif 10, was only located in ZmSSL5 and ZmSSL6 (Figure 2). ZmSSL7 and ZmSSL4 were from different groups, but the motif they contained was the same (Figure 2), probably due to those genes originating from the same branch (Figure 1). Some *ZmSSL* genes contained different motifs while some included the same motifs, suggesting that the functions of these genes may be different or redundant (Figure 2). Overall, these results indicated that the same group of *ZmSSL* genes shows a similar structure and this is consistent with the phylogenetic results presented in Figure 1.

### 2.4. Chromosomal Localization and Collinearity Analysis of ZmSSL Genes

**Figure 3:**
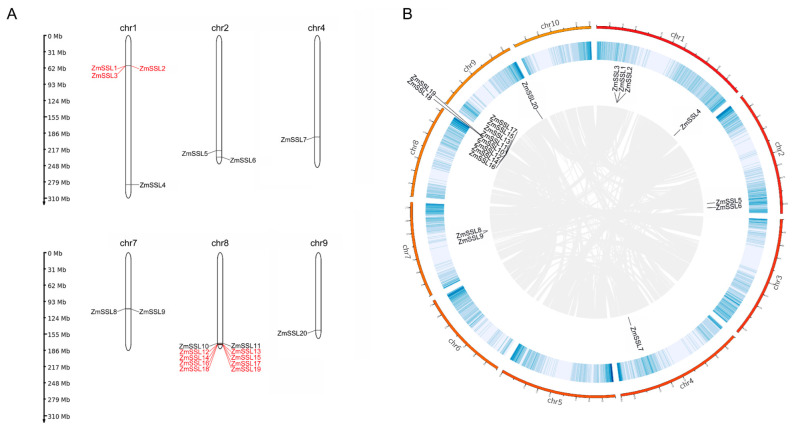
Chromosomal distribution and collinearity analysis of *ZmSSL* genes. (**A**) Twenty *ZmSSL* genes located in six chromosomes. Red represents tandem repeat. The scale bar on the left shows the mega base (Mb). (**B**) The schematic diagram of the relationship of *ZmSSL* genes between chromosomes. The heatmap in the inside circles indicates chromosome gene density. The gray lines in the background indicate collinear gene pairs in the maize genome.

Based on the annotation of the maize genome, we mapped the physical location of *ZmSSL* genes on maize chromosomes. As shown in Figure 3A, *ZmSSL1–3* located in Chr 1 and *ZmSSL12–19* located in Chr 8 were both tandem repeats. Further collinearity analysis of the duplication events of the *ZmSSL* gene using MCScanXs showed that no segmental duplications (homologous genes) were obtained (Figure 3B). These results indicated that tandem duplication is an important event in the evolution of the maize *SSL* gene family.

**Figure 4:**
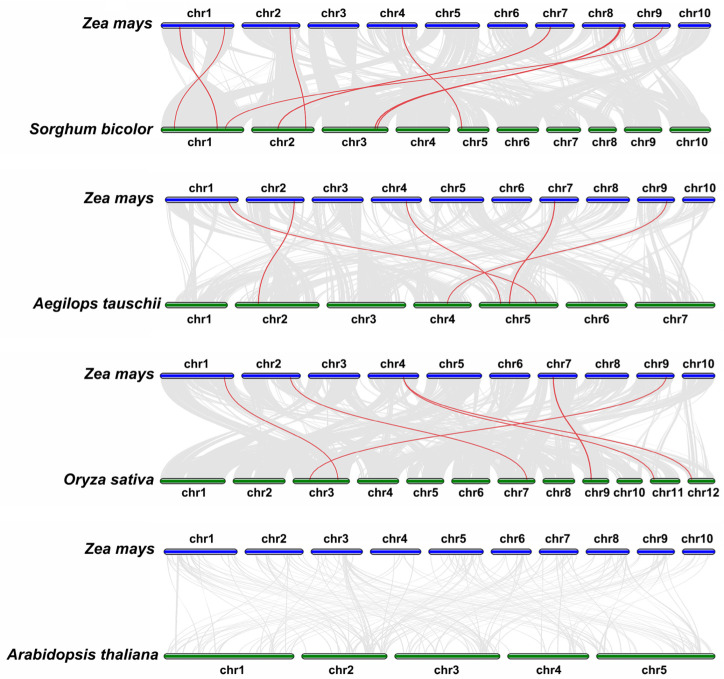
The collinearity diagram of *SSL* genes between maize and four other plants (sorghum, rice, *Ae. tauschii*, and Arabidopsis). The gray line in the background represents the collinear gene pairs between the two species, and the collinear genes are connected with a red line.

To further evaluate the evolutionary relationship of *ZmSSL* genes, a covariance analysis was performed. A total of 19 homologous pairs were identified between maize and sorghum, rice, *Ae. tauschii*, and Arabidopsis (8, 6, 5, and 0 collinear genes, respectively) (Figure 4). This indicates that the *SSL* family is conserved during the evolution process of explored grass crops.

### 2.5. The Cis-Elements Analysis of ZmSSL Gene Promoter Regions

**Figure 5:**
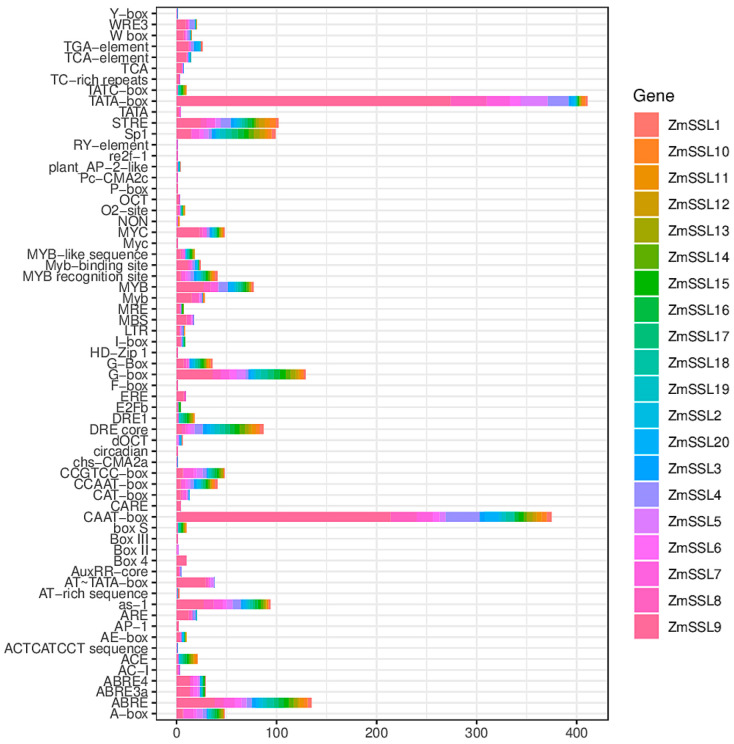
Predicted *cis*-elements in promoter regions of *ZmSSL* genes. A promoter region of about 2-kb upstream of *ZmSSL* genes are used for analysis. Different colors represent different genes. The *cis*-element counts are listed in Supplementary Table S3.

Using PlantCARE to analyze the promoter regions of the *ZmSLL* genes, we identified a large number of *cis*-elements involved in the development and hormones/abiotic/biotic stress responses in the promoter regions of most *ZmSSL* genes (Figure 5, Supplementary Table S3). These included light- and development-relative elements (CAT-box, HD-Zip1, CIRCADIAN, G-box, AE-box, CCAAT-box, etc.), ABA-responsive motifs (ABRE and ABRE4/3a), salicylic acid-relative elements (TCA-element), IAA response motifs (AuxRR-core and TGA-element), GA elements (P-box), MYB and MYC binding site (MBS and MYC) elements involved in drought response, TC-rich elements involved in defense and stress responses, low-temperature-responsive elements (LTR and WRE3), and STRE for antioxidant responses. Overall, these results suggested that *ZmSSL* genes may participate in maize growth, development, and responses to a stressful environment.

### 2.6. Expression Patterns of ZmSSL Genes in Different Maize Tissues under Normal Conditions

**Figure 6:**
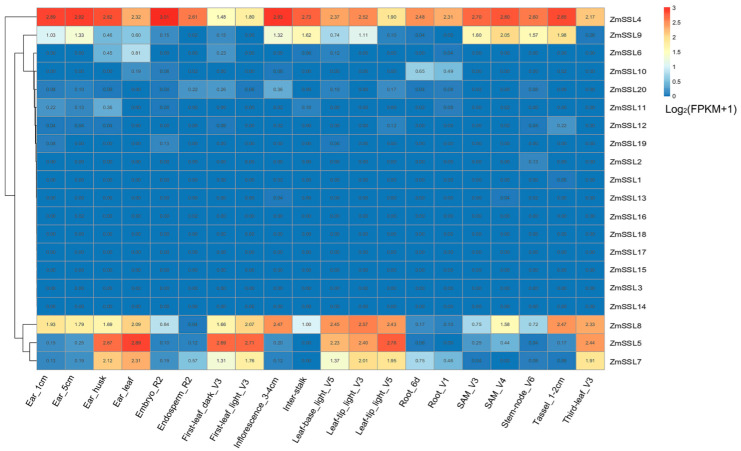
Tissue-specific expressions of *ZmSSL* genes. The heatmap construction is based on the fragments per kilobase per million mapped reads (FPKM) values of published RNA-seq data. Different tissues of maize are labeled in the figure. SAM: shoot apical meristem. Data links are shown in Supplementary Table S4.

To explore the expression patterns of *ZmSSL* genes in maize tissues under normal conditions, published transcriptome sequencing data [16] (Supplementary Table S4) from 15 different maize tissues (defined by different developmental periods), including ear, embryo, endosperm, stalk, leaf, root, shoot apical meristem (SAM), and tassel, were used to construct an expression heat map (Figure 6). The expression level of *ZmSSL4–9* genes was distinctive in different tissues (Figure 6). *ZmSSL4–6* belonging to Group II displayed higher expression in the reproductive organs (ear, ear-leaf, and embryo) (Figure 6), suggesting these genes may be important for maize reproductive growth. Two Group III members (*ZmSSL8* and *9*) showed the highest expression in the leaf-tip and SAM (Figure 6), revealing that these genes may be involved in maize nutrient growth. One Group II member, *ZmSSL20*, and all 13 Group IV members (*ZmSSL1–3*, *10–19*) showed almost no expression levels in any tissues, indicating that these genes may be involved in stress responses.

### 2.7. Expression Patterns of ZmSSL Genes under Abiotic/Biotic Stress Conditions

**Figure 7:**
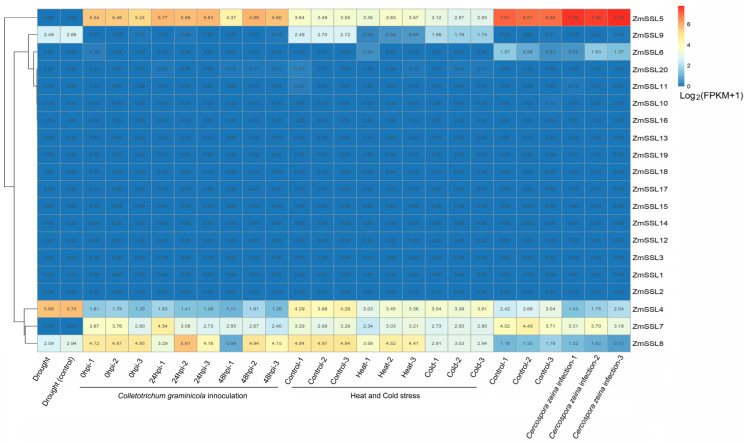
The heat map of *ZmSSL* genes under abiotic and biotic stresses. Different stresses are labeled within the figure. The data (except drought stress) were processed from three replicates of published transcriptomic data (Supplementary Table S5). Heatmap construction is based on the fragments per kilobase per million mapped reads (FPKM) values.

To explore the expression patterns of *ZmSSL* genes in maize tissues under abiotic and biotic stresses, published transcriptome sequencing data (Supplementary Table S5) for drought [17], heat or cold [18], and two fungal infections (*Colletotrichum graminicola* and *Cercospora zeina*) [19,20] were used to analyze and build an expression heat map (Figure 7). None of the 20 *ZmSSL* genes were induced during 1 h of dehydration stress (Figure 7), even though the promoter region was rich in drought-responsive elements (Figure 5 and Figure 7). *ZmSSL5* was induced after 24 h of *C. graminicola* infection (Figure 7), indicating it may be involved in maize responses to fungal infection. Both 50 °C for 4 h and 5 °C for 16 h clearly downregulated the expression of *ZmSSL4*, *ZmSSL5*, and *ZmSSL7–9* (Figure 7). *ZmSSL4* was downregulated by *C. zeina* infection while ZmSSL5 was upregulated, implying that it had the opposite effect in *C. zeina* infections (Figure 7). Similar to the tissue expression results (Figure 6), none of the 13 Group IV members or *ZmSSL20* responded to abiotic/biotic stresses, indicating these genes may be responsive to other stresses or are otherwise pseudogenes.

### 2.8. RT-qPCR Validation of ZmSSL Genes under Abiotic/Biotic Stresses

**Figure 8:**
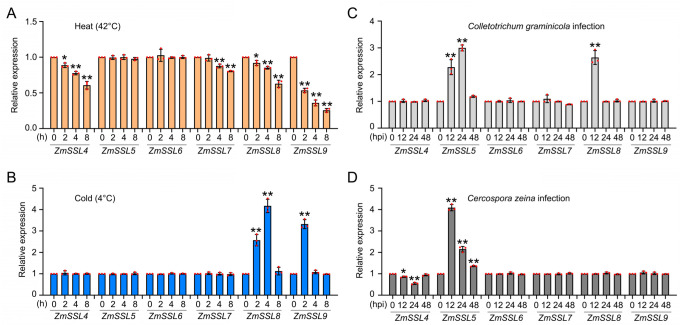
RT-qPCR analysis of the expression of *ZmSSL4–9* genes in maize under heat (**A**), cold (**B**), *Colletotrichum graminicola* (**C**), and *Cercospora zeina* (**D**) infection stresses. Treatment times and conditions are labeled within the figure. Values are mean ± SD; *n* = 3. ** *p* < 0.01, * *p* < 0.05 compared with control (Student’s *t* test).

To further analyze the expression levels of *ZmSSL* genes under abiotic/biotic stresses, we treated maize B73 to heat, cold, *C. graminicola*, and *C. zeina* stresses, and performed an RT-qPCR to explore the transcript level of *ZmSSL4–9*. The expression of four genes (*ZmSSL4*, *7*, *8*, *9*) was notably downregulated at 42 °C (Figure 8A). *ZmSSL8* and *ZmSSL9* both positively responded to cold stress (Figure 8B). The results after *C. graminicola* infection indicated that *ZmSSL5* and *ZmSSL8* were upregulated after 24 and 48 h post-inoculation (hpi), suggesting they may be involved in the response process (Figure 8C). Like the RNA-seq data (Figure 7), *ZmSSL4* and *ZmSSL5* displayed the opposite expression pattern under *C. zeina* infection (Figure 8D). Overall, six *ZmSSL* genes showed a diverse expression model under different biotic/abiotic stresses and may have different functions during these different stresses.

### 2.9. Subcellular Localization of ZmSSL4 and ZmSSL5

**Figure 9:**
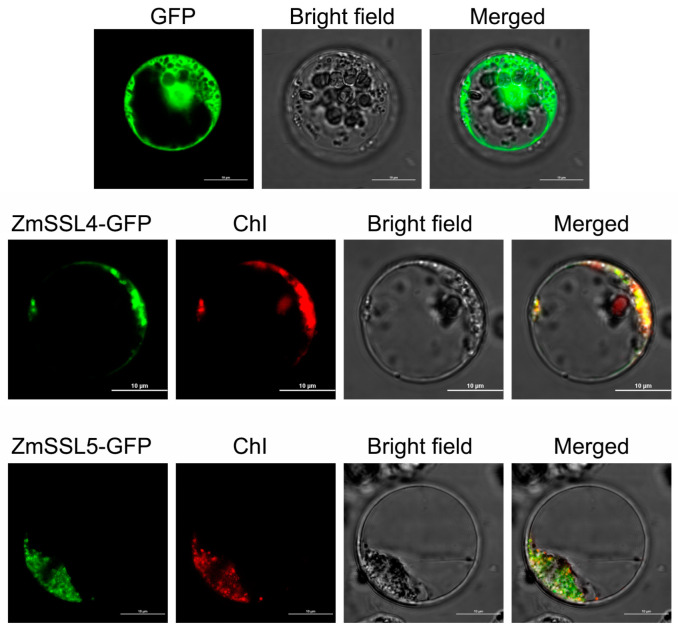
Subcellular localization of ZmSSL4 and ZmSSL5. The *GFP* or *ZmSSL4/5:GFP* fusion expression vectors were transformed into maize B73 mesophyll protoplasts using the PEG method. The GFP and chlorophyll autofluorescence (ChI) signals are labeled green and red, respectively. A bright field image is shown in the third column. The overlay is shown in the fourth column. The empty *GFP* vector was used as a control. GFP: green fluorescent protein. Bars = 10 μm.

To further explore the functions of the *ZmSSL* gene family, *ZmSSL4* and *ZmSSL5*, which exhibited opposite response patterns under *C. zeina* infection, were chosen to be evaluated in terms of their subcellular localization. As shown in Figure 9, in contrast to the localization of GFP control in maize mesophyll protoplasts, the ZmSSL4-GFP and ZmSSL5-GFP signals both overlapped with the fluorescence of chloroplasts (ChI), indicating that both ZmSSL4 and ZmSSL5 were located in chloroplasts.

## 3. Discussion

The *SSL* gene family is thought to be playing a significant role in plant responses to biotic/abiotic stresses [8,9]. Although many types of *SSL* genes have been identified in various plants through bioinformatics analysis, such as Arabidopsis [13], *Catharanthus roseus* [10,21], and *Solanum lycopersicum* [15], no relevant reports about the family membership and function analysis of *ZmSSL* genes have been undertaken in maize and other crops (sorghum, rice, and *Ae. tauschii*). In the current study, using AtSSLs and the conserved HMM motif of SSL protein as a query to blast genome data, a total of 20, 21, 16, and 33 *SSL* genes were identified in maize, sorghum, rice, and *Ae. tauschii*, respectively (Table 1 and Supplementary Table S2). *ZmSSL* genes were unevenly distributed on chromosomes 1, 2, 4, 7, 8, and 9 (Supplementary Table S1 and Figure 3A). Meanwhile, the family members of the other three plants were also unevenly distributed on chromosomes (Supplementary Table S1) indicating that the chromosomal distribution of *SSL* genes may be evolutionarily consistent in different species. Replication events play an important role in the amplification of gene family members during plant evolution; if the spacing between two homologous genes is less than five genes it is called a tandem repeat, and if the spacing between two homologous genes is greater than five genes, it is called segmental repeat [22,23,24]. In the present study, *ZmSSL1–3* located in chromosome 1 and *ZmSSL12–19* in chromosome 8 were both tandem repeats and no segmental repeat was founded in the maize genome (Figure 3A,B). Meanwhile, both *ZmSSL1–3* and *ZmSSL12–19* showed almost no expression under normal or stress conditions (Figure 6, Figure 7, Figure 8 and Figure 9), suggesting that tandem repeats played a significant role in expanding family members in the evolution of maize *SSL* genes and might be leading to a loss of gene function.

To understand the evolutionary relationship of *ZmSSL* genes, we used 105 SSL sequences from maize, sorghum, rice, *Ae. tauschii*, and Arabidopsis (15 members) to build a phylogenetic tree. The results showed that these *SSL* genes are divided into five groups (Figure 1). Except for Group V, each group contained *ZmSSL* genes, with Group IV having the highest number (13 members). TBtools were used to analyze the structure of the *ZmSSL* genes and the conserved motif/domain of amino acid sequences. As shown in Figure 2, *ZmSSL* genes in the same evolutionary group contained similar numbers of exons, introns, and protein motifs, which showed high correlations with the evolutionary analysis results. A recent study indicated that the *SSL* family members of *Populus trichocarpa* (13 members), *Arabidopsis thaliana*, and *Salix purpurea* (22 members) are divided into four groups based on the neighbor-joining (NJ) method of constructing a phylogenetic tree, and each group includes both *SSL* genes from three species, indicating that those from herbaceous and woody *SSL* genes might undergo relatively consistent evolutionary progress [8]. In our work, only Group I and Group II contained SSL genes from all five plants and all *AtSSL* genes were mainly included in Group I (Figure 1). This result suggested that *SSL* genes from Arabidopsis and crops may undergo different evolutionary patterns. Further cross-species collinearity analysis revealed that maize shared more homologous pairs with sorghum, rice, and *Ae. tauschii* than with Arabidopsis (Figure 4), suggesting that the *SSL* family in our explored crops is more conserved during evolution.

The analysis of *cis*-acting elements in promoters can provide a basis for the functional study of genes [25]. Existing studies have shown that there is an important correlation between the *cis*-acting elements of the promoter region and the expression level of specific genes [26,27]. Multiple *cis*-acting elements involved in plant growth, development, hormones, and abiotic/biotic stress response have been found in the promoter region of most *ZmSSL* genes (Figure 5 and Supplementary Table S3), suggesting that *ZmSSL* genes might play a significant role in maize adaptations to the environment. Based on the predicted results of *cis*-acting elements, we used published transcriptome data [17,18,19,20] (Supplementary Tables S4 and S5) (including different maize tissues and different abiotic/biotic stresses) to evaluate the expression pattern of 20 *ZmSSL* genes, and the results showed that the Group IV maize *SSL* gene members were not expressed in different tissues and were not significantly induced by stress treatment (Figure 6 and Figure 7). The gene density analysis of chromosomes indicated that the Group IV *ZmSSL* genes both showed tandem repeats in the genome (Figure 1 and Figure 3A). Perhaps multiple copy events during evolution led to the promoter regions of these genes being disrupted, resulting in a loss of expression of these *ZmSSL* genes during maize growth and development. The other six *ZmSSL* members displayed diverse expression patterns in different tissues, and some of them were positively and negatively induced by heat, cold, and infection with *C. graminicola* and *C. zeina* fungus (Figure 6 and Figure 7), indicating that *ZmSSL4–9* may be playing different roles in maize development and stress responses. Further RT-qPCR analysis confirmed that *ZmSSL4*, *7*, *8*, and *9* were downregulated by heat, while *ZmSSL8* and *ZmSSL9* were upregulated by cold (Figure 8). *ZmSSL5* and *ZmSSL8* both positively responded to the *C. graminicola* infection while *ZmSSL4* and *ZmSSL5* showed opposite expression levels during *C. zeina* infection (Figure 8). Heat and cold are the two main adverse factors leading to reduced maize production. *C. graminicola* leads to anthracnose in maize [28] and *C. zeina* causes maize gray leaf spot [29], both economically significant diseases worldwide. These results indicated that *ZmSSL* genes may play an important role in the growth and development of maize. However, the specific function of the *ZmSSL* genes needs to be further researched.

ZmSSL4, ZmSSL5, and AtSSL5–7 were both in Group II (Figure 1). A previous study showed that *AtSSL5–7* is positively modulated by SA, JA, and pathogen infection [9], while in this study, *ZmSSL4* and *ZmSSL5* displayed the opposite response under *C. zeina* stress (Figure 8). To further explore the function of *ZmSSL4/5*, we evaluated the localization of ZmSSL4 and ZmSSL5 in maize B73 mesophyll protoplasts. Based on the predicted results of subcellular protein localization, ZmSSL4 and ZmSSL5 were located in the plasma membrane and chloroplast/thylakoid, respectively (Table 1). As shown in Figure 9, both of these two proteins were located in chloroplasts. This result suggests that chloroplasts might the main place for *ZmSSL* genes to perform their function. This hypothesis needs to be confirmed through further investigation of *ZmSSL* genes in maize.

## 4. Materials and Methods

### 4.1. Plant Materials and Stress Treatments

The maize B73 inbred line was used in this research. Surface-sterilized B73 seeds were planted in nutrient soil and grown under a 16 h/8 h day/night photoperiod at 25 °C in a greenhouse. Two-week-old (V3 stage) maize seedlings were used to assess abiotic stress. For either heat or cold stress, the seedlings were put in a 42 °C or 4 °C growth chamber for 0, 2, 4, or 8 h. There were three replicates for each treatment and three pots of seedlings for each replicate. Two fungal pathogens, *Colletotrichum graminicola* and *Cercospora zeina*, were used to infect the leaf of B73 (V12 growth stage) with the brush method [19,20]. Inoculated (1 × 10^6^ conidia per mL) leaf material (two leaves per plant) was harvested at four time points (0 hdpi, 12 hdpi, 24 hdpi, and 48 hdpi) from three biological replicates. The fungus was prepared as described [19,20]. Leaves treated with water were used as controls. The maize leaves were harvested after stress treatments and then immediately frozen in liquid nitrogen and stored at −80 °C until use.

### 4.2. Identification of ZmSSL Genes

Arabidopsis thaliana SSL protein sequences (download in TAIR10) [13] and two conserved SSL structural domains PF03088 and PF20067 identified via the hidden Markov model (HMM) [8] were separately queried to be compared to *Zea mays* L. (https://www.ncbi.nlm.nih.gov/genome/?term=maize, accessed on 10 June 2023), *Sorghum bicolor* L. (https://www.ncbi.nlm.nih.gov/genome/?term=Sorghum, accessed on 10 June 2023), *Oryza sativa* L. (https://www.ncbi.nlm.nih.gov/genome/?term=Oryza+sativa, accessed on 10 June 2023), and *Aegilops tauschii* (https://www.ncbi.nlm.nih.gov/genome/?term=Aegilops+tauschii, accessed on 10 June 2023) genome annotate documents using BLASTP (e-value: 1 × 10^−5^ and identity threshold > 40%). The non-redundant protein sequences obtained from these two methods were further analyzed. The physical parameters and subcellular localization of SSL proteins were envaulted using the online ExPASy (https://web.expasy.org/protparam/, accessed on 12 June 2023) and WoLF PSORT (https://www.genscript.com/wolf-psort.html, accessed on 12 June 2023).

### 4.3. Phylogenetic and Structural Analysis of ZmSSL Genes

The identified SSL protein sequences from maize, sorghum, rice, *Ae. tauschii*, and Arabidopsis were aligned using Clustal X (Version 1.83) [30]. The aligned results were imported into MEGA7 (Version 7.0) to build a phylogenetic tree using the maximum likelihood method and 1000 bootstrap replicates. The conserved motifs of *ZmSSL* genes were evaluated with MEME (Version 5.5.0) [31] and visualized using TBtools v1.120 [32]. The exon–intron structures of *ZmSSL* genes were obtained and visualized with TBtools v1.120.

### 4.4. Chromosomal Localization and Collinearity Analysis

Genomic data downloaded from NCBI (see Section 4.2 for specific links) were used to map each *SSL* gene to its corresponding chromosomal location using MapGene2Chrom (http://mg2c.iask.in/mg2c_v2.1/; accessed on 21 August 2023) and TBtools v1.120. [32]. MCScan (Version X) (Multicollinearity Scanning Toolkit) [33] was used to determine the covariance relationships of *ZmSSL* genes or *SSL* genes from different species; the results were visualized using TBtools v1.120 [32].

### 4.5. Cis-Acting Element Analysis

The 2000 bp upstream of the transcription start site (TSS) sequence of each *ZmSSL* gene was extracted from the NCBI genome database. *Cis*-elements were predicted with PLACE (http://bioinformatics.psb.ugent.be/webtools/plantcare/html/, accessed on 3 August 2023) [34] and visualized with TBtools v1.120 [32].

### 4.6. Transcriptome Data Analysis

The RNA-seq data used for the tissue-specific expression analysis of *ZmSSL* genes under normal conditions were obtained in the published research [16]. The published RNA-seq data for heat/cold [18], drought [17], and fungal infection [19,20] were used to evaluate the ZmSSL gene expression levels under stress. Heatmap construction was based on the fragments per kilobase per million (FPKM) mapped reads values of published RNA-seq data and visualized with TBtools [32]. Different periods of tissues and stresses are labeled in Supplementary Tables S4 and S5.

### 4.7. RT-qPCR Analysis of Gene Expression

Total leaf RNA extraction was performed following a published protocol [35]. Total RNA (1 μg) was used for first-strand cDNA synthesis using a cDNA Kit (CWBIO, Beijing, China). The products were diluted with RNase-free water and used for the template. The RT-qPCR Mix (20 μL) included 6 μL cDNA, 2 μL RNase-free water, a total of 2 μL of each primer, and 10 μL SYBR Mix (Thermo Fisher Scientific, Waltham, MA, USA). A PCR was performed on a CFX96 Real-Time System (Bio-Rad, Hercules CA, USA) and the procedure was as follows: 95 °C for 5 min, followed by 44 cycles of denaturation at 95 °C for 15 s, annealing at 60 °C for 30 s, then melting curves were performed using 65–95 °C in the final step. *ZmActin1* (GRMZM2G126010) was used as the internal control. All primers used in the RT-qPCR are listed in Supplementary Table S6.

### 4.8. Subcellular Localization

For the subcellular localization of *ZmSSL4* and *ZmSSL5*, the CDS of ZmSSL4 and ZmSSL5 were amplified without the stop codon using primers ZmSSL4-F/R and ZmSSL5-F/R (Supplementary Table S6). Amplicons were ligated into the pROKII plasmid [35,36]. An empty pROKII vector was used as a control. *ZmSSL4/5-GFP* plasmid DNA (20 μg) was transformed into maize mesophyll protoplasts following a published protocol [35,37]. The protoplasts were incubated at 25 °C for 17 h. The GFP and chlorophyll autofluorescence signals were observed with a scanning confocal microscope (Andor Revolution WD, https://andor.oxinst.com/).

### 4.9. Statistical Analysis

Statistical analysis was performed using SPSS 19.0. Significance (*p* < 0.05; *p* < 0.01) was assessed by using the Student’s *t* test.

## 5. Conclusions

In this study, 20 *SSL* genes were identified in maize through homologous alignment. Their chromosomal localization, phylogenetic relationship, gene and protein structure, *cis*-elements of promoter regions, and expression patterns under normal or stress conditions were further analyzed. The 20 *ZmSSL* genes were unevenly distributed on chromosomes. The phylogenetic analysis indicated that *ZmSSL* genes were located in four groups and members in each group exhibited the same gene and protein structure. Tandem duplications may be the main reason leading to the increasing copy number of *ZmSSL* genes in the maize genome, which may be leading to gene functional redundancy or no function. Moreover, *ZmSSL* genes had a high degree of conservatism during the evolution of our explored gramineae crops (sorghum, rice, and *Ae. tauschii*). Furthermore, six members of the *ZmSSL* genes, *ZmSSL4–9*, displayed wide expression in different tissues under normal conditions and were positively or negatively modulated by abiotic (heat and cold) and biotic (*C. graminicola* and *C.* *zeina* infection) stresses. This work may provide useful information to further evaluate the biological functions of *ZmSSL* genes.

## Figures and Tables

**Table 1:** Characteristics of *SSL* genes in maize.

Gene NCBI ID	Gene Name	CDS (bp)	Protein Size (aa)	MW (kDa)	pI	Subcellular Protein Location
XM_008657285.2	*ZmSSL1*	1017	338	36.31	6.08	Extracellular/Cell wall
XM_008657302.2	*ZmSSL2*	1020	339	36.46	5.89	Extracellular/Cell wall
XM_008657311.2	*ZmSSL3*	1017	338	36.33	6.08	Extracellular/Cell wall
NM_001156536.2	*ZmSSL4*	1197	398	44.10	6.34	Plasma membrane
NM_001148541.1	*ZmSSL5*	1104	367	39.46	5.25	Chloroplast/Thylakoid
XM_008673664.2	*ZmSSL6*	654	217	22.64	4.53	Chloroplast/Thylakoid
NM_001139223.1	*ZmSSL7*	1173	390	41.94	8.92	Extracellular/Cell wall
NM_001157297.1	*ZmSSL8*	1032	343	36.17	7.73	Chloroplast/Thylakoid
XM_008654423.3	*ZmSSL9*	1074	357	38.25	7.75	Chloroplast/Thylakoid
XM_008658790.4	*ZmSSL10*	1038	345	36.66	6.00	Chloroplast/Thylakoid
NM_001157473.1	*ZmSSL11*	1038	345	36.63	5.81	Chloroplast/Thylakoid
XM_008659695.3	*ZmSSL12*	594	197	20.81	7.73	Chloroplast/Thylakoid
XM_008658833.3	*ZmSSL13*	1038	345	36.78	7.01	Chloroplast/Thylakoid
XM_008658834.2	*ZmSSL14*	1038	345	36.74	6.61	Chloroplast/Thylakoid
XM_008658837.2	*ZmSSL15*	1038	345	36.81	6.61	Chloroplast/Thylakoid
XM_008658836.3	*ZmSSL16*	1038	345	36.78	7.02	Chloroplast/Thylakoid
XM_020542371.1	*ZmSSL17*	1050	349	37.09	7.01	Chloroplast/Thylakoid
XM_008658839.2	*ZmSSL18*	1050	349	37.05	6.09	Chloroplast/Thylakoid
XM_008658840.2	*ZmSSL19*	1038	345	36.75	7.01	Chloroplast/Thylakoid
NM_001324231.1	*ZmSSL20*	1239	412	46.54	6.00	Endoplasmic reticulum

## Data Availability

The data presented in this study are available on request from the corresponding author.

## Supplementary Materials

The supporting information can be downloaded at: https://www.mdpi.com/article/10.3390/ijms241914733/s1.
