# Supplementary material for: The Genome-Wide Identification, Characterization, and Expression Analysis of the Strictosidine Synthase-like Family in Maize (Zea mays L.)
Source: Int J Mol Sci. 2023 Sep 29;24(19):14733. doi: 10.3390/ijms241914733 (PMC10572891; doi:10.3390/ijms241914733)
Supplement: Supplementary file 1 [file ijms-24-14733-s001.zip › Supplementary Table S1.pdf]

Supplementary Table S1. Chromosome location of *SSL* genes in maize, sorghum, *A. tauschii*, rice.

Gene NCBI ID	Gene name	Chromosome location	
XM_008657285.2	*ZmSSL1*	chr1 57765926	57767063
XM_008657302.2	*ZmSSL2*	chr1 57839451	57840473
XM_008657311.2	*ZmSSL3*	chr1 57931853	57933018
NM_001156536.2	*ZmSSL4*	chr1 283339547	283343836
NM_001148541.1	*ZmSSL5*	chr2 217816869	217819033
XM_008673664.2	*ZmSSL6*	chr2 230318018	230319097
NM_001139223.1	*ZmSSL7*	chr4 192686880	192690390
NM_001157297.1	*ZmSSL8*	chr7 106494786	106497772
XM_008654423.3	*ZmSSL9*	chr7 106530695	106547501
XM_008658790.4	*ZmSSL10*	chr8 172251596	172253051
NM_001157473.1	*ZmSSL11*	chr8 172317613	172318880
XM_008659695.3	*ZmSSL12*	chr8 174314286	174315473
XM_008658833.3	*ZmSSL13*	chr8 174331242	174332515
XM_008658834.2	*ZmSSL14*	chr8 174358455	174359659
XM_008658837.2	*ZmSSL15*	chr8 174395363	174396627
XM_008658836.3	*ZmSSL16*	chr8 174410231	174411557
XM_020542371.1	*ZmSSL17*	chr8 174430134	174431217
XM_008658839.2	*ZmSSL18*	chr8 174482109	174483408
XM_008658840.2	*ZmSSL19*	chr8 174500075	174501305
NM_001324231.1	*ZmSSL20*	chr9 147078558	147080384
XM_002463826.2	*SbSSL1*	chr1 6859543	6862526
XM_002467521.2	*SbSSL2*	chr1 59691570	59693299
XM_002467825.2	*SbSSL3*	chr1 66394286	66395456
XM_021450900.1	*SbSSL4*	chr1 70741147	70742951
XM_002460105.2	*SbSSL5*	chr2 57083619	57085977
XM_002460106.2	*SbSSL6*	chr2 57098251	57108121
XM_021454025.1	*SbSSL7*	chr2 73117634	73119617
XM_021454024.1	*SbSSL8*	chr2 73138055	73140079
XM_002456214.2	*SbSSL9*	chr3 61486680	61487971
XM_002456598.2	*SbSSL10*	chr3 67591011	67592753
XM_002456676.2	*SbSSL11*	chr3 68860446	68861672
XM_002452406.2	*SbSSL12*	chr4 56536269	56537510
XM_002452874.1	*SbSSL13*	chr4 65410858	65411898
XM_002452931.2	*SbSSL14*	chr4 66353384	66354807
XM_002450198.2	*SbSSL15*	chr5 2609045	2613027
XM_002448917.2	*SbSSL16*	chr5 2668432	2672828
XM_002443544.2	*SbSSL17*	chr8 61143309	61144998
XM_021466141.1	*SbSSL18*	chr8 61167632	61177583
XM_002440687.2	*SbSSL19*	chr9 7859462	7860031
XM_002438595.2	*SbSSL20*	chr10 52416523	52418163

XM_002438796.1	*SbSSL21*	chr10	56908806	56909846
XM_020328930.3	*AetSSL1*	chr1	299082938	299085192
XM_020311481.1	*AetSSL2*	chr2	33638849	33639978
XM_020311479.3	*AetSSL3*	chr2	33676626	33678709
XM_020311480.3	*AetSSL4*	chr2	33680203	33681635
XM_020332098.1	*AetSSL5*	chr2	71431862	71432803
XM_020345579.3	*AetSSL6*	chr2	122004608	122006651
XM_020331117.3	*AetSSL7*	chr2	122299282	122301235
XM_020321132.3	*AetSSL8*	chr3	367134471	367135904
XM_020321538.2	*AetSSL9*	chr3	603545210	603546491
XM_045233917.1	*AetSSL10*	chr3	604261675	604263216
XM_020322447.3	*AetSSL11*	chr4	377349425	377351945
XM_020317747.3	*AetSSL12*	chr5	229164106	229167108
XM_020312210.3	*AetSSL13*	chr5	314403773	314405607
XM_020312214.3	*AetSSL14*	chr5	314642224	314645598
XM_020324203.2	*AetSSL15*	chr5	465855619	465857107
XM_020293478.3	*AetSSL16*	chr5	473098767	473101358
XM_040394268.2	*AetSSL17*	chr7	43894988	43896165
XM_020331968.3	*AetSSL18*	chr7	43960967	43963227
XM_020326298.1	*AetSSL19*	chr7	44048675	44049175
XM_020326303.1	*AetSSL20*	chr7	44058399	44059436
XM_020291800.2	*AetSSL21*	chr7	90228398	90230558
XM_020308049.1	*AetSSL22*	chr7	90298207	90299253
XM_020304825.3	*AetSSL23*	chr7	90508588	90510185
XM_020327805.3	*AetSSL24*	chr7	90612354	90613780
XM_040397558.1	*AetSSL25*	NW_025487989.1	5	681
XM_020335935.2	*AetSSL26*	NW_025495645.1	16	762
XM_040397573.1	*AetSSL27*	NW_025500911.1	7	831
XM_040397578.1	*AetSSL28*	NW_025504744.1	8	914
XM_040397587.1	*AetSSL29*	NW_025507561.1	6	921
XM_020292326.2	*AetSSL30*	NW_025512283.1	660	1049
XM_040397643.1	*AetSSL31*	NW_025517689.1	5	1356
XM_045232141.1	*AetSSL32*	NW_025531641.1	8092	12923
XM_020292328.1	*AetSSL33*	NW_025533088.1	38744	39781
XM_015774752.2	*OsSSL1*	chr3	8664268	8666379
XM_015777851.1	*OsSSL2*	chr3	30927228	30929818
XM_015786906.2	*OsSSL3*	chr6	25080840	25081930
XM_015786907.2	*OsSSL4*	chr6	25085509	25094294
XM_015790531.2	*OsSSL5*	chr7	21518901	21520060
XM_026027054.1	*OsSSL6*	chr7	21558382	21559551
XM_015790533.1	*OsSSL7*	chr7	21564056	21573587
XM_015789067.2	*OsSSL8*	chr7	25287500	25290041

XM_015793705.2	*OsSSL9*	chr8	4370774	4372947
XM_015794103.2	*OsSSL10*	chr8	21549609	21551813
XM_015755842.2	*OsSSL11*	chr9	12454357	12460991
XM_015755680.2	*OsSSL12*	chr9	12466343	12484831
XM_015755936.2	*OsSSL13*	chr9	12536480	12540746
XM_015757236.2	*OsSSL14*	chr10	21224291	21226900
XM_015761176.2	*OsSSL15*	chr11	1986272	1988401
XM_015764132.2	*OsSSL16*	chr12	1882224	1888271
