# Supplementary material for: The Genome-Wide Identification, Characterization, and Expression Analysis of the Strictosidine Synthase-like Family in Maize (Zea mays L.)
Source: Int J Mol Sci. 2023 Sep 29;24(19):14733. doi: 10.3390/ijms241914733 (PMC10572891; doi:10.3390/ijms241914733)
Supplement: Supplementary file 1 [file ijms-24-14733-s001.zip › Supplementary Table S2.pdf]

Supplementary Table S2. Characteristics of *SSL* genes in sorghum, *Ae. tauschii*, rice, and Arabidopsis.

Gene NCBI ID	Gene name	CDS (bp)	Protein Size (aa)	MW (kDa)	pI	Subcellular Location
			**Sorghum**			
XM_002463826.2	*SbSSL1*	1197	398	44.27	6.34	Plasma membrane
XM_002467521.2	*SbSSL2*	1101	366	39.96	5.92	Extracellular/Cell wall
XM_002467825.2	*SbSSL3*	1026	341	36.69	6.53	Chloroplast/Thylakoid
XM_021450900.1	*SbSSL4*	1272	423	48.07	8.51	Endoplasmic reticulum
XM_002460105.2	*SbSSL5*	1053	350	37.06	6.98	Chloroplast/Thylakoid
XM_002460106.2	*SbSSL6*	1101	366	39.38	8.99	Mitochondrion
XM_021454025.1	*SbSSL7*	1083	360	38.76	5.16	Chloroplast/Thylakoid
XM_021454024.1	*SbSSL8*	1107	368	39.75	5.18	Extracellular/Cell wall
XM_002456214.2	*SbSSL9*	1044	347	36.86	8.63	Chloroplast/Thylakoid
XM_002456598.2	*SbSSL10*	1038	345	36.84	5.80	Chloroplast/Thylakoid
XM_002456676.2	*SbSSL11*	1044	347	36.75	5.89	Chloroplast/Thylakoid
XM_002452406.2	*SbSSL12*	1035	344	36.67	9.03	Vacular membrane
XM_002452874.1	*SbSSL13*	1041	346	37.27	9.12	Vacular membrane
XM_002452931.2	*SbSSL14*	1041	346	37.34	9.02	Vacular membrane
XM_002450198.2	*SbSSL15*	1173	390	43.04	5.97	Endoplasmic reticulum
XM_002448917.2	*SbSSL16*	1179	392	43.03	6.28	Endoplasmic reticulum
XM_002443544.2	*SbSSL17*	1032	343	37.99	6.00	Chloroplast/Thylakoid
XM_021466141.1	*SbSSL18*	984	327	35.56	8.78	Chloroplast/Thylakoid
XM_002440687.2	*SbSSL19*	570	189	19.94	7.09	Chloroplast/Thylakoid
XM_002438595.2	*SbSSL20*	1164	387	40.41	8.31	Endoplasmic reticulum
XM_002438796.1	*SbSSL21*	1041	346	37.25	9.12	Vacular membrane
			Ae. tauschii			
XM_020328930.3	*AetSSL1*	1104	367	39.28	5.05	Extracellular/Cell wall
XM_020311481.1	*AetSSL2*	1032	343	36.57	6.40	Mitochondrion
XM_020311479.3	*AetSSL3*	1155	384	41.58	5.75	Cytosol
XM_020311480.3	*AetSSL4*	1032	343	36.90	7.09	Extracellular/Cell wall
XM_020332098.1	*AetSSL5*	942	313	34.32	5.98	Vacular membrane
XM_020345579.3	*AetSSL6*	1104	367	38.97	4.97	Endoplasmic reticulum
XM_020331117.3	*AetSSL7*	1098	365	38.98	5.72	Endoplasmic reticulum
XM_020321132.3	*AetSSL8*	1074	357	38.61	6.55	Chloroplast/Thylakoid
XM_020321538.2	*AetSSL9*	1170	389	42.55	8.85	Cytosol
XM_045233917.1	*AetSSL10*	1170	389	42.53	8.90	Cytosol
XM_020322447.3	*AetSSL11*	1242	413	46.71	7.18	Endoplasmic reticulum
XM_020317747.3	*AetSSL12*	1125	374	40.87	6.28	Chloroplast/Thylakoid
XM_020312210.3	*AetSSL13*	1041	346	37.20	7.76	Chloroplast/Thylakoid
XM_020312214.3	*AetSSL14*	1095	364	39.48	8.19	Chloroplast/Thylakoid
XM_020324203.2	*AetSSL15*	1014	337	36.33	6.91	Chloroplast/Thylakoid
XM_020293478.3	*AetSSL16*	1197	398	43.64	5.91	Extracellular/Cell wall
XM_040394268.2	*AetSSL17*	1035	344	37.84	8.69	Vacular membrane
XM_020331968.3	*AetSSL18*	1086	361	39.52	7.71	Chloroplast/Thylakoid

XM_020326298.1	*AetSSL19*	501	166	18.62	9.35	Cytosol
XM_020326303.1	*AetSSL20*	1038	345	37.67	8.24	Vacular membrane
XM_020291800.2	*AetSSL21*	1047	348	37.72	8.90	Chloroplast/Thylakoid
XM_020308049.1	*AetSSL22*	1047	348	37.77	9.30	Chloroplast/Thylakoid
XM_020304825.3	*AetSSL23*	1038	345	37.48	8.98	Chloroplast/Thylakoid
XM_020327805.3	*AetSSL24*	1050	349	37.85	9.16	Chloroplast/Thylakoid
XM_040397558.1	*AetSSL25*	675	225	24.51	5.96	Partial sequence
XM_020335935.2	*AetSSL26*	747	248	27.06	6.81	Cytosol
XM_040397573.1	*AetSSL27*	825	274	29.75	6.82	Chloroplast/Thylakoid
XM_040397578.1	*AetSSL28*	789	262	28.40	8.67	Vacular membrane
XM_040397587.1	*AetSSL29*	840	280	31.05	8.99	Partial sequence
XM_020292326.2	*AetSSL30*	390	129	14.32	9.89	Cytosol
XM_040397643.1	*AetSSL31*	912	304	33.52	7.23	Partial sequence
XM_045232141.1	*AetSSL32*	1170	389	42.55	9.04	Cytosol
XM_020292328.1	*AetSSL33*	1038	345	37.71	9.37	Vacular membrane
			**Rice**			
XM_015774752.2	*OsSSL1*	1284	427	47.80	6.25	Cytosol
XM_015777851.1	*OsSSL2*	1443	480	53.71	8.75	Cytosol
XM_015786906.2	*OsSSL3*	1038	345	37.55	9.57	Chloroplast/Thylakoid
XM_015786907.2	*OsSSL4*	1044	347	37.62	9.47	Chloroplast/Thylakoid
XM_015790531.2	*OsSSL5*	1053	350	37.42	8.80	Cytosol
XM_026027054.1	*OsSSL6*	747	248	26.98	9.15	Cytosol
XM_015790533.1	*OsSSL7*	1080	359	38.68	9.05	Cytosol
XM_015789067.2	*OsSSL8*	1104	367	39.66	4.91	Chloroplast/Thylakoid
XM_015793705.2	*OsSSL9*	1071	356	38.06	6.15	Mitochondrion
XM_015794103.2	*OsSSL10*	1053	350	38.04	8.13	Extracellular/Cell wall
XM_015755842.2	*OsSSL11*	1095	364	38.19	8.53	Extracellular/Cell wall
XM_015755680.2	*OsSSL12*	1101	366	38.51	6.56	Chloroplast/Thylakoid
XM_015755936.2	*OsSSL13*	1149	382	40.01	9.16	Cytosol
XM_015757236.2	*OsSSL14*	1110	369	39.51	5.25	Extracellular/Cell wall
XM_015761176.2	*OsSSL15*	1116	371	41.19	8.80	Cytosol
XM_015764132.2	*OsSSL16*	2013	670	73.69	9.44	Plasma membrane
			**Arabidopsis**			
NM_129694.5	*AtSSL1*	1185	394	44.39	6.21	Endoplasmic reticulum
NM_129693.4	*AtSSL2*	1131	376	41.48	6.20	Extracellular/Cell wall
NM_100720.3	*AtSSL3*	1173	390	44.04	8.11	Cytosol
NM_115001.3	*AtSSL4*	1113	370	41.60	5.65	Chloroplast/Thylakoid
NM_115002.4	*AtSSL5*	1116	371	41.64	6.20	Chloroplast/Thylakoid
NM_115003.3	*AtSSL6*	1116	371	41.38	5.83	Chloroplast/Thylakoid
NM_115004.5	*AtSSL7*	1116	371	41.12	5.40	Chloroplast/Thylakoid
NM_115560.4	*AtSSL8*	1131	376	41.98	5.78	Extracellular/Cell wall
NM_115561.5	*AtSSL9*	1113	370	41.46	6.56	Vacular membrane
NM_115562.4	*AtSSL10*	1125	374	41.00	7.71	Extracellular/Cell wall
NM_106059.5	*AtSSL11*	990	329	34.67	9.66	Chloroplast/Thylakoid

NM_106061.4	*AtSSL12*	1008	335	35.29	5.60	Extracellular/Cell wall
NM_202733.3	*AtSSL13*	1212	403	45.63	6.41	Cytosol
NM_147884.6	*AtSSL14*	978	325	34.18	8.27	Extracellular/Cell wall
NM_106060.3	*AtSSL15*	1188	395	44.53	6.52	Extracellular/Cell wall
