# Supplementary material for: The Genome-Wide Identification, Characterization, and Expression Analysis of the Strictosidine Synthase-like Family in Maize (Zea mays L.)
Source: Int J Mol Sci. 2023 Sep 29;24(19):14733. doi: 10.3390/ijms241914733 (PMC10572891; doi:10.3390/ijms241914733)
Supplement: Supplementary file 1 [file ijms-24-14733-s001.zip › Supplementary Table S6.pdf]

Supplementary Table S6. List of Primers

Primer name	Primer sequence
ZmActin1-F	TACGAGATGCCTGATGGTCAGGTCA
ZmActin1-R	TGGAGTTGTACGTGGCCTCATGGAC
ZmSSL4-RT-F	TCGCAACCTCCAATTTCCC
ZmSSL4-RT-R	CCTTCTCGCCTTTCAACCAG
ZmSSL5-RT-F	TTCTGGATTGCCGTCCTCC
ZmSSL5-RT-R	CCCTTGCTCCACTCGCTGA
ZmSSL6-RT-F	AGGGTCGGTGACGAGGGAGT
ZmSSL6-RT-R	GCTGGCGTCGCTGAAGTAGA
ZmSSL7-RT-F	TCTCCTACCCCTGGCTCCG
ZmSSL7-RT-R	TCCCGTCCTCCTCGCTCA
ZmSSL8-RT-F	TTCGCCTACAACTCCAAGCACA
ZmSSL8-RT-R	CGTAAAGGTCGCCGGACTGC
ZmSSL9-RT-F	CCAGCGGAGCGACTACATCA
ZmSSL9-RT-R	CCGTTGGGGAAGGACAGG
ZmSSL4-F	AGAACACGGGGGACTCTAGA ATGGCGTCGCCGGG
ZmSSL4-R	CTCACCATGGTACCCTCGAG AGACGCCTTCGCGAGGTC
ZmSSL5-F	AGAACACGGGGGACTCTAGA ATGGCGCCTGGCCTCC
ZmSSL5-R	CTCACCATGGTACCCTCGAG CGAAGATACTGCTCCCTGCTTC
